# Taking down the FLAG! How Insect Cell Expression Challenges an Established Tag-System

**DOI:** 10.1371/journal.pone.0037779

**Published:** 2012-06-06

**Authors:** Peter M. Schmidt, Lindsay G. Sparrow, Rebecca M. Attwood, Xiaowen Xiao, Tim E. Adams, Jennifer L. McKimm-Breschkin

**Affiliations:** CSIRO Materials Science and Engineering, Parkville, Victoria, Australia; Deutsches Krebsforschungszentrum, Germany

## Abstract

In 1988 the preceding journal of *Nature Biotechnology*, *Bio/Technology*, reported a work by Hopp and co-workers about a new tag system for the identification and purification of recombinant proteins: the FLAG-tag. Beside the extensively used hexa-his tag system the FLAG-tag has gained broad popularity due to its small size, its high solubility, the presence of an internal Enterokinase cleavage site, and the commercial availability of high-affinity anti-FLAG antibodies. Surprisingly, considering the heavy use of FLAG in numerous laboratories world-wide, we identified in insect cells a post-translational modification (PTM) that abolishes the FLAG-anti-FLAG interaction rendering this tag system ineffectual for secreted proteins. The present publication shows that the tyrosine that is part of the crucial FLAG epitope DYK is highly susceptible to sulfation, a PTM catalysed by the enzyme family of Tyrosylprotein-Sulfo-transferases (TPSTs). We showed that this modification can result in less than 20% of secreted FLAG-tagged protein being accessible for purification questioning the universal applicability of this established tag system.

## Introduction

With high-throughput sequencing and ready-to-use gene synthesis becoming more and more routine for all laboratories, the focus for the efficient production of recombinant proteins has shifted towards facilitating the expression and subsequent purification of the encoded proteins. To allow efficient purification and to overcome known problems of protein production such as aggregation, inefficient translation, limited solubility, or degradation, affinity tag systems have become an indispensable tool [1]. Affinity tags allow single step purification procedures resulting in highly pure protein. In addition, tags can promote proper folding, reduce aggregation, or increase solubility thereby increasing the yields of fused recombinant proteins. Beside the omnipresent hexa-his tag alternative tag systems have been developed over the years all with different strengths and weaknesses. From these non-his-tag-systems (e.g. MBP, GST, CBP, STREP, myc, FLAG [1]) the FLAG tag is one of the most commonly used systems.

FLAG was initially described by Hopp and co-workers in 1988 [2] and its sequence DYKDDDDK was designed based on the following assumptions: 1. The tag should be as short as possible but still long enough to form an epitope for antibody recognition; 2. It should be highly soluble to be exposed on the surface of any fused protein minimizing its impact on protein folding; 3. The sequence DDDDK was selected to allow enterokinase cleavage of the tag; 4. Lysine (K) in the third position was introduced to increase hydrophilicity; and 5. Tyrosine (Y) was selected as aromatic residues often improve antibody binding [2]. The first antibody used to purify FLAG-tagged proteins (M1; clone 4E11) was shown to be Ca^2+^-dependent allowing the mild elution of bound proteins via EDTA [3], [4]. However, while the Ca^2+^-dependency remains controversial [5], the constraint that the FLAG-tag had to be at the N-terminus and not be preceded by other amino acids fostered the development of further anti-FLAG mAbs, namely M2 and M5. These allowed more flexibility with respect to the positioning of the tag. Due to this versatility and the availability of a hybridoma cell line, M2 has become the most widely used anti-FLAG mAb, despite various companies have recently introduced new anti-FLAG antibodies (for review see [6]). Although there have been several attempts to optimize the FLAG- sequence via ELISA [7] or phage display [8] the original FLAG sequence DYKDDDDK is still used for virtually all FLAG-tagged proteins.

Surprisingly, considering the ubiquitous use of FLAG in numerous laboratories world-wide, the present publication describes an unobserved post-translational modification (PTM) of this tag that abolishes the FLAG-anti-FLAG interaction and renders this system ineffective for the detection or purification of secreted proteins. Our results clearly show that the tyrosine, that is part of the crucial FLAG epitope DYK, is highly susceptible to tyrosine sulfation, a PTM catalyzed by the enzyme family of Tyrosine-Protein-Sulfo-Transferases (TPSTs) in the trans-Golgi network. As membrane proteins are processed via the same cellular pathway, the FLAG-anti-FLAG detection might be also impaired for these proteins. In some cases less than 20% of the expressed protein was able to be purified questioning the universal applicability of this tag system.

## Results

**Figure 1:**
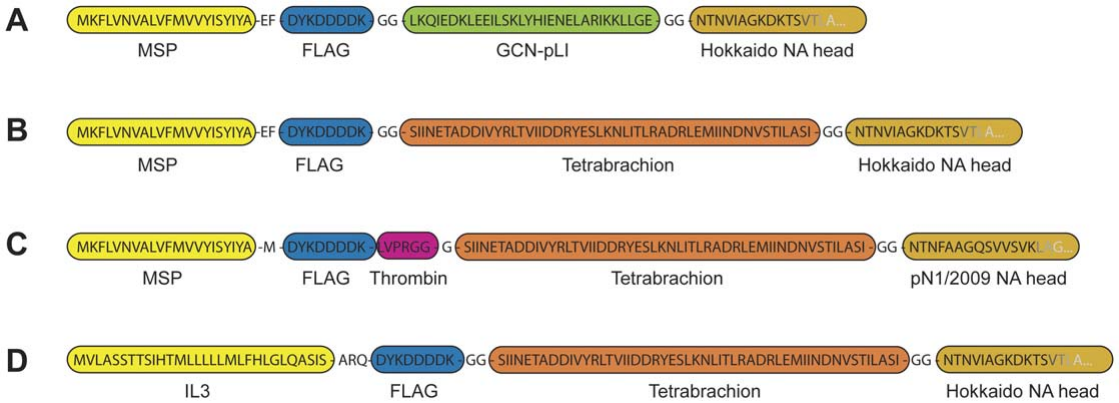
Design of the different NA expression constructs. The figure shows the constitution and partial sequence of the different NA expression constructs used to express secreted NA: Recombinant Hokkaido H1N1 NA with an artificial stalk based on the Yeast transcription factor GCN-pLI (A) or the Tetrabrachion tetramerization domain of Staphylothermus marinus (B); The third construct is based on the Tetrabrachion stalk fused to the NA head sequence of pN1/2009 NA. It contains a Thrombin cleavage site C-terminal of the FLAG allowing the efficient cleavage of the tag. Constructs A to C use the Melittin signal peptide (MSP) to drive secretion of the respective NA. Figure 1D shows the sequence of the construct used to express Hokkaido H1N1 NA in mammalian cells. This construct uses the mouse Interleukin 3 (IL3) secretion signal to drive protein secretion.

**Figure 2:**
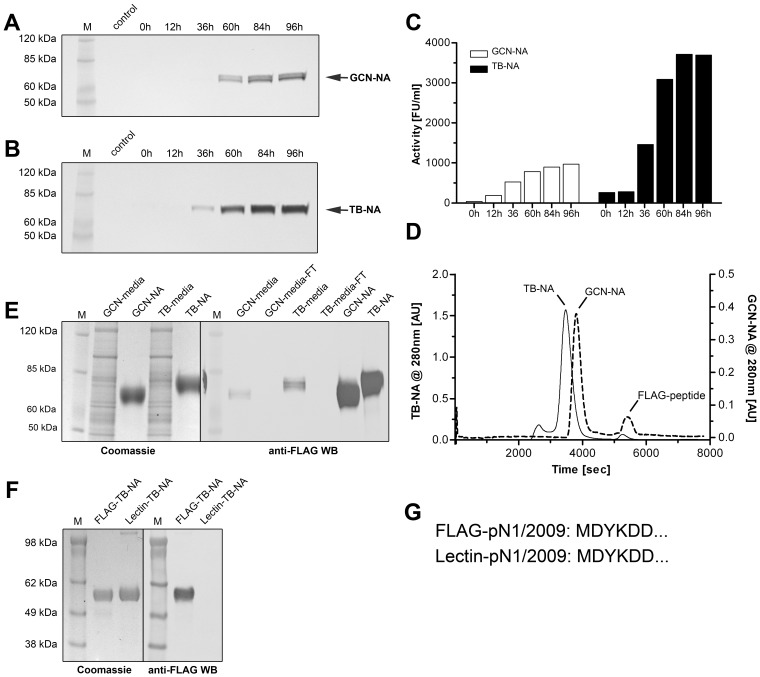
Expression and purification of recombinant NA in insect cells. The expression and secretion of GCN-pLI and TB-based soluble Hokkaido NA was monitored by anti-FLAG WB (2A, B) and by NA activity assays (2C). The gel filtration chromatogram for both NA constructs is shown in 2D. 2E shows the processed insect cell media before and after anti-FLAG affinity purification in a Coomassie stained gel (2E left panel) and corresponding WB (2E right panel). Figure 2F shows the FLAG- and Lectin/IEX-purified pN1/2009 NA loaded onto an SDS-PAGE and stained by Coomassie (2F left panel) or detected by anti-FLAG WB (2F right panel). The amino acid sequence obtained by N-terminal sequencing of both enzymes is shown in 2G.

In order to obtain purified neuraminidase (NA) for biochemical characterization and crystallization studies human N1 NA containing the artificial GCN-pLI or the Tetrabrachion stalks (Fig. 1A, B) were expressed as described earlier [9]. Both insect cell expressions showed maximum NA secretion 84 h post infection without visible degradation products as judged by anti-FLAG western blot (WB; Fig. 2A, B). The Tetrabrachion-based construct (Fig. 2B) resulted in higher yields in agreement with the corresponding NA activity assays (Fig. 2C) which showed approximately four-fold higher NA activity for the Tetrabrachion-based NA compared to the GCN-pLI-NA. The higher expression levels of the TB-based NA-construct as well as its higher molecular weight were corroborated by gel filtration chromatography showing a four-fold higher absorption and faster elution compared to GCN-pLI-NA (Fig. 2D). Both expressions resulted in highly pure NA with no visible contaminating proteins as judged by SDS-PAGE (Fig. 2E left panel) and anti-FLAG WB (Fig. 2E right panel). The flow-through after anti-FLAG affinity purification showed no signal in the anti-FLAG WB suggesting that the entire FLAG-reactive NA has been purified from the media in a single run (Fig. 2E, right panel). Surprisingly, when the flow-throughs were checked for residual NA activity it became evident that 49% of the activity of the GCN-pLI-based enzyme and 84% of the Tetrabrachion-based NA (data not shown) were still in the flow-through despite the results of the WB suggesting the entire depletion of both expressed enzymes. Similar results were obtained for the TB-based pN1/2009 construct (Fig. 1C; data not shown).

As these results indicated that in some cases more than 80% of the expressed NA was not purified or even detected by the anti-FLAG antibody, the reason for the failure of the FLAG-affinity purification was further investigated. As the FLAG-tag was located at the N-terminus of the proteins (Fig. 1) unexpected cleavage of the artificial stalks or N-terminal truncation of its essential DYK epitope [7] while not affecting NA activity might have explained the inability to purify these enzymes. To identify the potential cleavage or truncation site non-FLAG-reactive TB-NA (pN1/2009) was purified via lentil-lectin affinity chromatography followed by gel filtration [10] and ion exchange chromatography (see Fig. S1, S2). The purified NA was of similar purity as the FLAG-purified NA, judged by Coomassie stained SDS PAGE (Fig. 2F, left panel) but entirely non-reactive towards the anti-FLAG mAb in the corresponding WB (Fig. 2F, right panel). Subsequent N-terminal sequencing showed surprisingly that both enzymes had a fully functional N-terminal FLAG tag (Fig. 2G) ruling out any unexpected cleavage or N-terminal truncation of the stalk as reason for the failure of the FLAG-anti-FLAG system.

**Figure 3:**
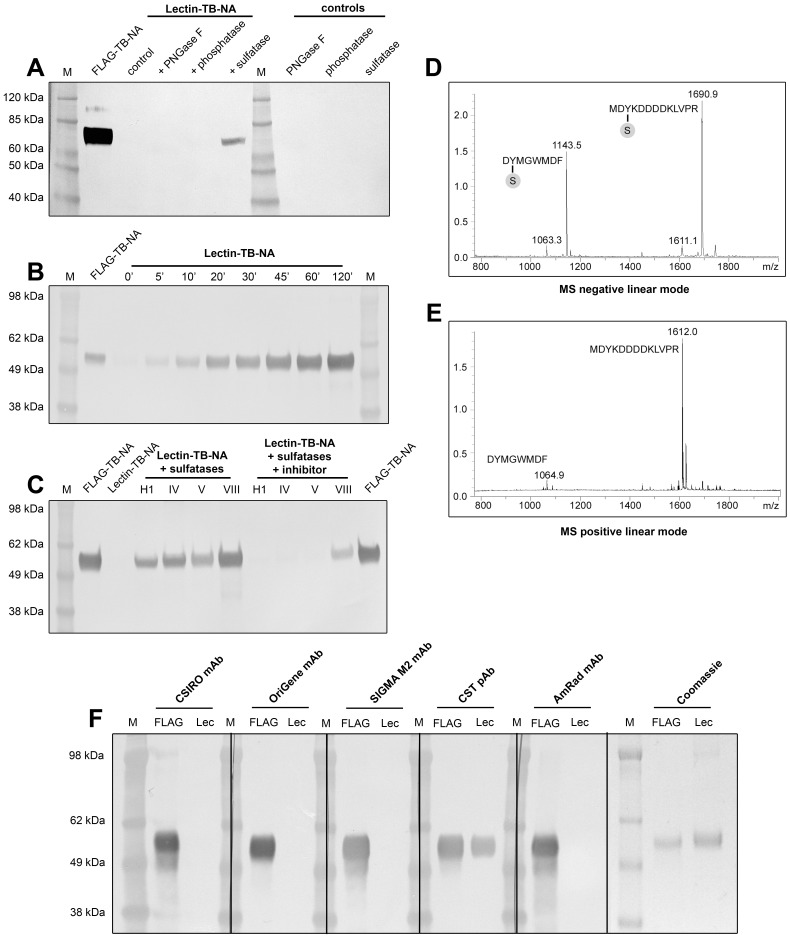
Identification of FLAG-tag tyrosine-sulfation. Lectin purified TB-Hokkaido NA was incubated with different PTM-cleaving enzymes. The anti-FLAG WB is shown in Fig. 3A. Addition of sulfatase made the FLAG tag accessible to the anti-FLAG mAb. Desulfation kinetics of Lectin/IEX-purified pN1/2009 NA using sulfatase type VIII (Abalone entrails) are shown in 3B. Activity of all 4 sulfatases tested was efficiently inhibited by the addition of vanadate-phenyl-Ester (3C). Fig. 3D shows the negative linear mode MS of the sulfated control peptide CCK-8 at 1143.5 Da and its non-sulfated form at 1063.3 Da (−80 Da). The Thrombin cleaved FLAG tag was detected in its sulfated and non-sulfated form at a molecular weight of 1690.9 and 1611.1 Da (−80 Da), respectively. Fig. 3E shows the same sample measured in positive linear mode. Only the non-sulfated peptides (−80 Da) were detected with an additional weight due to protonation (+1 Da). Fig. 3F shows the WB of FLAG- and Lectin-purified NA (pN1/2009) detected with different anti-FLAG antibodies as well as the Coomassie stained gel of the same samples.

These results narrowed down potential explanations for the non-reactivity of the tag to unfavorable folding or post-translational modification of the tag. As the Tetrabrachion stalk has been described as extremely rigid [11], [12] an accidental folding of the highly soluble FLAG tag masking the epitope in only a certain subpopulation of the expressed protein appeared rather unlikely. In contrast, preliminary in-silico prediction suggested a potential phosphorylation or sulfation of the FLAG tyrosine as well as a glycosylation of an adjacent asparagine residue (www.expasy.ch). From these predictions, tyrosine sulfation appeared to be most likely as this PTM is a known modification of secreted proteins [13], [14] and it requires a high density of negative charged residues in close proximity to the tyrosine (a comprehensive review on the characteristics of the sulfation consensus site can be found here [15]). To test these predictions, non-FLAG reactive TB-N1 (Hokkaido) was incubated with λ-phosphatase, H1 sulfatase, or PNGaseF and subsequently subjected to anti-FLAG WB analysis. The results of these blots showed clearly that incubation with sulfatase but not with phosphatase or PNGaseF restored the reactivity of the FLAG epitope for the anti-FLAG antibody (Fig. 3A) suggesting that indeed tyrosine-sulfation of the FLAG tag was responsible for masking the FLAG epitope. Subsequent experiments showed that desulfation of the FLAG tag could be accomplished by different sulfatases including type H1 (Helix pomatia), type IV and V (Patella vulgata) and type VIII (Abalone entrails). All four sulfatases tested were efficiently blocked by the potent sulfatase inhibitor vanadate-phenyl-ester [16] underlining the specificity of the desulfation reaction (Fig. 3C). In addition the time-dependency of the desulfation reaction was shown (Fig. 3B).

Although all strings of evidence indicated that the FLAG tag is rendered inaccessible by tyrosine sulfation the direct identification of the FLAG sulfo-tyrosine was key as proof of the hypothesis. Mass spectrometry (MS), which has become in invaluable tool for the identification of PTMs, can be run in positive or negative mode in order to analyze positively (protonated) or negatively (deprotonated) charged ions. As protonation of the tyrosine-sulfate has been shown to induce the loss of the sulfate group, positive mode MS is not able to detect this PTM. In contrast, negative mode MS can detect tyrosine sulfation if carefully optimized [17], [18], [19]. In order to directly show the sulfation of the FLAG tag, non-FLAG reactive pN1/2009 NA was purified as described in the Experimental Procedures section. This construct has an additional thrombin cleavage site (Fig. 1C) that allows the removal of the tyrosine-sulfated FLAG-tag for subsequent MS analysis (see supplemental Fig. S3). The sample was spiked with sulfated CCK-8 peptide (DY(SO_3_)MGWMDF, 1143.35 Da) as an intrinsic control to show the stability of sulfo-tyrosine under the reaction conditions. Following cleavage, the reaction mixture was subjected to size exclusion chromatography and the resulting fractions were directly analyzed by MS. Fig. 3D shows the CCK-8 peptide with a molecular weight of 1143.5 Da in its sulfated form whereas only a minor fraction of desulfated CCK-8 (1063.3 Da) was observed suggesting that sulfo-tyrosine containing peptides remain stable under these experimental conditions. The second large MS peak had a molecular weight of 1690.9 Da in good agreement with the predicted molecular mass of the sulfated FLAG-tag including the residues of the Thrombin cleavage site (MDY(SO_3_)KDDDDKLVPR). As seen for CCK-8 only a minor fraction of the cleaved FLAG peptide was detected in its desulfated form at 1611.1 Da (Fig. 3D; Table 1). Increasing the MS laser power output resulted in a relative shift from the sulfated to the desulfated form of both peptides (data not shown). Running the MS in positive linear mode lead to the loss of the sulfated form of the tyrosine for both peptides (Fig. 3E) resulting in a molecular weights of 1064.9 Da and 1612.0 Da, respectively. The additional weight of 1 Da compared to negative mode MS can be attributed to the protonation of the desulfated peptides.

**Table 1:** Theoretical and experimental peptide masses.

	Sulfation status	Amino acid Sequence	Theoretical molecular weight	Experimental molecular weight (negative mode)	Experimental molecular weight (positive mode)
**FLAG tag+ Thrombin site**	sulfated (S)	MDY**(SO_3_H)**KDDDDKLVPR	1689.70 Da (S)	1690.9 Da (S) 1611.1 Da (NS)	not detected 1612.0 Da (NS)
	non-sulfated (NS)	MDYKDDDDKLVPR	1609.80 Da (NS)	not measured	not measured
**CCK-8 peptide**	sulfated (S)	DY**(SO_3_H)**MGWMDF	1143.35 Da (S)	1143.5 Da (S) 1063.3 Da (NS)	not detected 1064.9 Da (NS)
	non-sulfated (NS)	DYMGWMDF	1063.40 Da (NS)	not measured	not measured

The Thrombin-cleaved sulfated FLAG tag was purified from the remaining proteins (NA and Thrombin) by using a peptide column as described in the Materials and Methods section. Purified peptides were then subjected to negative and positive mode MS.

After tyrosine sulfation was established as being responsible for masking the FLAG epitope, the reactivity of different commercial anti-FLAG antibodies (Table 2) towards sulfated FLAG was evaluated. The inability to detect sulfated FLAG-tag was not limited to the anti-FLAG mAb produced in-house. Three commercially available monoclonal antibodies (SIGMA anti-FLAG M2, OriGene anti-DDK, AmRad anti-FLAG) failed to detect the sulfo-FLAG (Fig. 3F). In contrast, the polyclonal anti-FLAG antibody from Cell Signaling Technology (CST) reacted with sulfated FLAG-tagged NA although signal strength was reduced (Fig. 3F, Table 2).

**Table 2:** Anti-FLAG antibodies used to detect FLAG-tagged NA.

Supplier	Antibody name	Type	Source	Order number	Reactivity towards FLAG	Reactivity towards Sulfo-FLAG
**CSIRO (in-house)**	Anti-FLAG	mAb	mouse	-	strong	none
**OriGENE**	Anti-DDK	mAb	mouse	TA50011	strong	none
**SIGMA**	Anti-FLAG M2-HRP	mAb	mouse	A8592	strong	none
**Cell Signaling Technology**	DYKDDDDK tag antibody	pAb	rabbit	2368	strong	partial
**AmRad**	Anti-FLAG	mAb	mouse	-	strong	none

The table lists the five anti-FLAG antibodies used in the present work. The Anti-FLAG mAb from AmRad is no longer commercially available.

**Figure 4:**
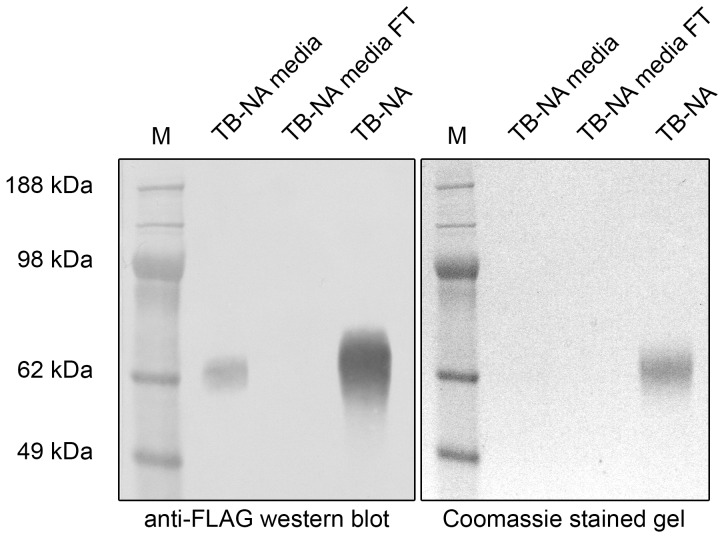
Expression and purification of recombinant NA in mammalian cells. Human TB-NA (Hokkaido) fused to the Tetrabrachion stalk (Fig. 1B) was cloned into the pApex-3 vector and expressed in HEK293T cells. Media, flow-through, and purified NA were loaded onto SDS-PAGE and detected by anti-FLAG WB (left panel) or stained by Coomassie (right panel). Secreted FLAG-reactive NA was detected by anti-FLAG WB. Based on the anti-FLAG WB the column flow-through did not contain any residual FLAG reactive NA.

As it would be laborious and cost-intensive for many laboratories to replace an established tag system including vectors, antibodies, and affinity matrices we tried to decrease the likelihood of FLAG sulfation. This PTM is catalyzed by the enzyme family of TPSTs which rely on a consensus sequence to identify potential sulfation sites [15]. Although the composition of the consensus sequence is not entirely understood [15], the NA expression vectors were modified to express different residues N-terminal of the FLAG tag to test if FLAG sulfation could be decreased. Changing the sequence AEF-DYKDDDK (Fig. 1A, B) to M-DYKDDDDK (Fig. 1C) did not result in any change of the sulfation ratio for expressed TB-NA (data not shown). However, enzymes expressed with a blunt N-terminal FLAG tag were virtually entirely depleted from the media by anti-FLAG affinity chromatography suggesting that the shortened sequence was not longer recognized as a substrate by TPSTs. In parallel, it was investigated whether the expression of FLAG-tagged NA in a mammalian cell line (HEK 293T) would result in a more favorable ratio of sulfated to non-sulfated FLAG-tagged NA compared to expression in insect cells (BVES). The sequence encoding for the human seasonal TB-N1 was cloned into a mammalian expression vector and expressed in HEK293T cells as described in the Experimental Procedures section. After transient transfections, the media showed strong NA activity and the secreted NA could be detected by anti-FLAG WB (Fig. 4). However, after affinity purification of the FLAG-tagged NA, there was virtually no residual NA detectable in the column flow-through either by activity assays (data not shown) or by anti-FLAG WB (Fig. 4), suggesting purification of the entire FLAG-tagged NA.

## Discussion

Since its initial description by Hopp and co-worker in 1988 [2] the FLAG tag has been used for numerous applications due to its small size, its high solubility, the availability of high-affinity anti-FLAG mAbs (and the respective hybridoma cell lines), and the possibility of cleaving the tag with enterokinase. Over the last 20 years FLAG has been used for a broad variety of protein detection and purification strategies including immuno-precipitation, protein-purification, and tracking of tagged proteins in cells, tissues or even genetically modified mice [20]. The broad usage of the tag-system is also reflected by a strong commercial interest. Various companies offer a broad portfolio of FLAG-mAb-based products such as affinity matrices, coated microtiter plates, or coated magnetic beads (Table 2). Surprisingly, considering the ubiquitous use of FLAG in numerous laboratories world-wide, the present publication describes a yet unobserved PTM of this tag that abolishes the FLAG-anti-FLAG interaction and renders this system ineffective for the detection or purification of secreted proteins. Our results clearly show that the FLAG tyrosine, which is part of the crucial FLAG epitope DYK, is highly susceptible to tyrosine sulfation, a PTM catalyzed by the enzyme family of Tyrosylprotein-sulfo-transferases (TPSTs) in the trans-Golgi network.

In agreement with earlier findings the expression and purification strategy resulted in highly pure and active NA [9]. However, when checking the column flow-through a surprisingly high residual NA activity was found indicating that the majority of secreted NA had not been purified by the FLAG affinity chromatography. As the high avidity of the tetrameric recombinant NA generally allows the complete depletion of FLAG-tagged NA from the media (as indicated by WB), this result suggested the existence of a non-FLAG-reactive sub-population of secreted NA. As many recombinant proteins are routinely detected by anti-tag mAbs (e.g. anti-FLAG, anti-hexa-his, anti-c-myc) the unexpected failure of a tag system like in this case could result in a dramatic reduction of purification yields. The fact that not only FLAG but also the commonly used hexa-his tag has been shown to undergo post-translational modification [21] underlines the necessity to double-check even established tag systems to avoid major losses during proteins purification. The general development towards ready-to-use expression kits including tagged expression vector and corresponding anti-tag mAbs aggravate this problem as it promises the user a trouble-free protein-purification without major need for optimization.

The fact that the sulfation of the FLAG tyrosine was not described earlier despite its frequent usage might be attributed to a combination of unfavorable characteristics of the tag: First, tyrosine sulfation in general is not as well known as other PTMs and has been described to be rather unstable [14], [15]; second the only commercially available mAb that detects sulfo-tyrosine independent from the peptide backbone doesn't detect sulfo-FLAG due to the adjacent positive charge of the lysine [22] and finally neither N-terminal sequencing nor standard positive mode MS [17] are able to detect this modification. In addition, the presence of a neighboring positive charge has been shown to stabilize sulfo-tyrosine [23] making sulfo-FLAG not only hard to detect but also unusually stable even under acidic conditions (data not shown).

The present data unequivocally identified sulfation of the FLAG tyrosine as being responsible for abolishing detection of the tag by all commercial anti-FLAG mAbs tested. Despite the FLAG sequence being designed to meet certain scientific needs [2], it accidently also represents a strong consensus site for tyrosine sulfation. Algorithms such as Sulfinator or Sulfo-site [15], [24] predict sulfation of the FLAG tyrosine with high likelihood. These predictions are further corroborated by published results showing that Coagulation factor VIII as a known tyrosine sulfated peptide, has a consensus sequence that is very closely related to FLAG (D^364^Y^365^DDD^368^ [25]). Addition of aryl-sulfatases from different species effectively removed the sulfate group restoring the FLAG-anti-FLAG interaction. However, as most commercially available sulfatase preparations are of limited purity and contain a broad variety of different enzymes, it could not have been excluded that the unmasking of the FLAG tag was catalyzed by a different enzyme rather than sulfatase. To address this point, phenyl-vanadate-ester was added to the desulfation reaction. Phenyl-vanadate-ester has been published to inhibit aryl-sulfatases with high affinity and specificity by imitating the substrate's transition state [26]. Addition of this compound effectively suppressed the unmasking of the FLAG tag indicating that indeed sulfation of the FLAG tyrosine has been responsible for the inability of the anti-FLAG mAb to detect the tag. All sulfatases were effectively blocked by phenyl-vanadate with the exception of sulfatase VIII from Abalone Entrails which showed some residual activity. This result is in agreement with published findings showing that this enzyme has a higher specific activity than other sulfatases tested [27].

A more direct detection of the sulfo-tyrosine by WB using a pan-sulfo-tyrosine mAb [22], [28] failed due to the fact that the FLAG sulfo-tyrosine is followed by a positively charged lysine which has been published to circumvent the detection of this PTM [22]. Electrostatic interaction between positively charged residues and the sulfate group has also been published to increase stability of sulfo-tyrosine [23]. This might explain the finding that even 24 h of incubation of sulfo-FLAG-NA with 10% TCA resulted in hardly any loss of the sulfate group from the FLAG tag (data not shown). Although all strings of evidence indicated tyrosine sulfation as being responsible for the inability of anti-FLAG mAbs to detect the tag, the direct proof of this PTM was key to proof this hypothesis. In order to achieve this goal, non-FLAG reactive NA was expressed, purified, and the presumably sulfated tag was cleaved by Thrombin. The subsequent negative mode MS showed clearly a peak of the expected molecular weight for sulfated FLAG. Furthermore, in agreement with published results, increasing the laser power, switching from linear to reflectron mode (data not shown), or switching from negative to positive detection mode decreased the molecular weight of the peak by 80 Da indicating the loss of the sulfate group [17]. These results proved also that the measured 80 Da increase in molecular weight was due to tyrosine-sulfation and not -phosphorylation as the latter is stable in positive mode MS.

After having established that the inability of the in-house anti-FLAG mAb to detect the tag was due to sulfation of the FLAG tyrosine, further commercially available anti-FLAG antibodies were screened. All anti-FLAG mAbs tested failed to detect sulfo-FLAG whereas the polyclonal anti-FLAG antibody from CST detected sulfo-FLAG although with reduced sensitivity (Table 2). As mAbs are generally selected for highest affinity, these results suggest that the DYK sequence represents one of the best epitopes for antibody recognition, supporting the initial hypothesis of Hopp and co-workers [2]. However, as the signal of the polyclonal Ab was only reduced but not abolished, it should be possible to develop anti-FLAG mAbs that are not affected by tyrosine sulfation.

Beside the identification of tyrosine sulfation as a potential problem for FLAG-based purification of recombinant proteins, the present data also suggests potential strategies to overcome this problem. The secretion of NA with an N-terminal FLAG tag without preceding residues (e.g. AEF, or M; see Fig. 1) dramatically reduced the amount of non-FLAG reactive NA (data not shown). This finding suggests that the shortened tag sequence is not longer accepted by TPST as substrate. Although this approach worked well it might not be feasible e.g. due to cloning strategies. Another possibility to increase the efficiency of FLAG-tag based protein purification could be the use of multiple FLAG tags as such as SIGMA's triple FLAG tag. Despite prediction algorithms such as SulfoSite indicate a similar probability for all three tyrosines to be sulfated it is unlikely that all DYK epitopes of the triple FLAG tag will be affected. By combining both approaches the purification yields of FLAG-tagged proteins might be strongly increased. Also to position the FLAG tag at the C- rather than the N-terminus of a protein of interest could reduce the probability of its sulfation by TPST. We haven't tested this potential approach as the introduction of a tag at the C-terminus of NA is more likely to affect the enzyme's catalytic activity and, in addition, SulfoSite predicts tyrosine sulfation of a potential C-terminal FLAG with comparable probability as for the N-terminus. However, it can't be excluded that the different position of the tag could reduce sulfation and therefore increase purification yields.

The drawback of all these strategies is that existing expression constructs need to be modified. Therefore, the results obtained with the HEK293T expression system were of more interest. Although TPST is conserved throughout virtually all eukaryotic organisms [14], the expressed N1 was entirely purified from the media in a single run indicating that the FLAG tag was not sulfated at all. This is even more remarkable as the N-terminal FLAG-tag in this construct is preceded by three residues. Whether the use of a different secretion signal (Interleukin vs. Mellitin) was responsible for this result needs to be clarified by future experiments. Alternatively, HEK293T cells might be deficient for TPST although the fact that similar results were obtained in CHO cells would argue against this explanation.

In summary, the present data shows that the FLAG tag, which has been used for more than two decades, can be heavily impaired if used for secreted and possibly membrane proteins due to the TPST catalyzed sulfation of the FLAG tyrosine which is crucial for antibody recognition. This finding impacts on any kind of FLAG-anti-FLAG interaction such as immuno-precipitation, localization of FLAG-tagged proteins, as well as protein purification. As anti-tag antibodies rather than antibodies against the protein of interest are routinely used to track, localize and identify recombinant proteins, the present observation might indicate that the FLAG-anti-FLAG system has failed elsewhere to accurately detect and bind expressed tagged proteins. This could be especially true for structural proteins which have no enzymatic activity that allows tracking the presence of non-FLAG-reactive protein. Although the observation why mammalian expression systems such as CHO or HEK293T cells did not sulfate the FLAG tag needs to be further investigated, it represents a promising result for researchers expressing FLAG-tagged proteins. However, the fact that the observed tag sulfation is affected by the sequence of the protein, the preceding residues as well as the expression strategy and cell system, is questioning the universal reliability of FLAG as tag system in general especially as a broad variety of alternative tag systems are available [1].

## Materials and Methods

### Materials, cells, media

4-Methylumbelliferyl N-acetyl-a-D-Neuraminic acid (MUNANA) was obtained from Carbosynth (Compton, Berkshire, UK). All other chemicals including 4-Methylumbelliferone (MU) were of analytical grade and purchased from Sigma-Aldrich (Castle Hill, Australia). FLAG peptide was ordered from Peptide2.0; Sulfo-CCK-8 peptide was obtained from Bachem (H-2080, Bubendorf, Switzerland). Thrombin and Sulfatases (type H-1, IV, V, VIII) were purchased from Sigma-Aldrich. NAs were purified in Tris buffer [25 mM] pH 6.5 containing 10 mM Ca2+, and 0.02% azide (TBA). For activity assays NAs were diluted in TBA containing BSA at a final concentration of 1 mg/ml.

### Construction of recombinant NA expression system

The construction, cloning, and generation of NA encoding baculovirus was performed as described elsewhere [9]. Two different NA sequences were expressed, namely a human seasonal influenza H1N1 NA from A/Hokkaido/15/02 (hereafter referred to as Hokkaido [29]) as well as a pandemic H1N1/09 influenza NA (hereafter referred to as pN1/2009) derived from a multisequence alignment of various pN1/2009 NA sequences. Both NA heads were fused to an artificial stalk based on the yeast transcription factor GCN4-pLI or the Tetrabrachion tetramerization domain from the deep sea archae bacterium *Staphylothermus marinus*. An N-terminal FLAG tag was used for detection and subsequent purification (Fig. 1, S4). The PCR products were cloned into the transfer vector pFastBac downstream of a coding sequence for the melittin signaling peptide (MSP). Bacmids and baculovirus particles were constructed using the Bac-to-Bac system (Invitrogen, Carlsbad, CA) according to the manufacturer's protocols. For the expression in HEK293T cells, a PCR fragment corresponding to the N-terminal FLAG tag - Tetrabrachion tetramerization domain - NA coding region was generated and cloned downstream of the mouse Interleukin-3 signal peptide sequence in the mammalian expression vector, pApex-3 [30].

### Expression of recombinant NAs

The recombinant NA-constructs were expressed as described elsewhere [9]. Briefly, Sf21 insect cells (Invitrogen) cultured in SF-900 II serum free medium (Invitrogen) were infected at a density of 2×10^6^/ml with a MOI of 1. Secreted NA activity in the media and cell viability were assessed every 24 h. Subsequently, supernatant was cleared (40,000 g, 1 h, 4°C) and filtered. Azide and the protease inhibitor E-64 [31] were added to a final concentration of 0.02% and 1 µM, respectively. For mammalian expression, human HEK293T cells were transiently transfected with vector DNA using FuGENE (Roche, Indianapolis, USA) according to the manufacturer's instructions. Briefly, 293T cells were seeded at 3×10^5^ cells per well of a six-well plate in 2 ml of Dulbecco's modified Eagle's medium (DMEM; Invitrogen) containing 10% fetal calf serum (FCS). After 24 hours, DNA∶lipid complexes (1 µg DNA; 3 µl FuGENE) were added and culture supernatants harvested 49–72 h later. Biosynthesis and secretion of FLAG-tagged NA was determined by WB as described below. To recover larger volumes of culture supernatant, transfections were performed in 10 cm^2^ culture dishes seeded with either 3×10^6^ HEK293T cells in DMEM/10% FCS. Transfections were performed with Lipofectamine 2000 (Invitrogen) according to the manufacturer's instructions. The next day, the culture supernatants were replaced with 15 ml of serum-free Freestyle 293 Expression Medium (Invitrogen). Culture supernatants were harvested after 72 h.

### Purification of recombinant NAs

Anti-FLAG affinity chromatography (Mini-Leak Low; Kem-En-Tec A/S, Copenhagen, Denmark) was used to purify FLAG-tagged NA from the supernatant. Bound NA was eluted by TBA buffer containing 0.25 mg/ml FLAG peptide (DYKDDDDK). FLAG-tagged NAs were further purified and concentrated as described [9]. Successful depletion of FLAG-reactive NA in the flow-through was verified by WB (see below). Flow-through without FLAG-reactivity was subjected to lentil lectin chromatography as previously described [10]. Briefly, the FLAG-depleted media was run through the lentil lectin affinity matrix (lentil lectin Sepharose 4B; GE Healthcare, Rydalmere, NSW, Australia) overnight at 4°C. Bound glyco-proteins were eluted with α-D-Methylmannoside [200 mM] in TBA, concentrated and subjected to gel filtration chromatography. If necessary, NA activity containing fractions were further purified by anion exchange chromatography (MonoQ; GE Healthcare).

### SDS-PAGE and western blotting

SDS-PAGE was performed using NuPAGE precast gels (Invitrogen) according to the manufacturer's protocols. Protein bands were visualized by Coomassie Brilliant Blue (NuSep, Frenchs Forest, NSW, Australia). WB were performed using the iBlot system (Invitrogen) according to the manufacturer's protocol. Equal sample loading and the quality of the PAGE and the subsequent WB transfer were routinely verified by Ponceau staining of the blotting membrane. Transferred FLAG-tagged proteins were visualized as described elsewhere [32].

### Desulfation of sulfo-FLAG

To desulfate NA samples prior to WB, purified non-FLAG reactive NA was incubated in acetate buffer [100 mM] pH 5.5 with different sulfatases at a ratio of 0.1 U sulfatase (sulfatase inhibition assay) or 1.0 U sulfatase per µg NA (desulfation kinetic) at 37°C for 3 h or as indicated. The reaction was stopped by boiling the sample with SDS-PAGE sample buffer. Sulfatase inhibition by vanadate-phenyl-ester was performed as described elsewhere [16]. Briefly, orthovanadate [400 µM] and phenol [40 mM] were mixed 1∶1 and incubated for 10 min to form the vanadate-phenyl-ester. Subsequently, the inhibitor was added to the sulfatase reaction in a dilution of 1/10 resulting in a final concentration of vanadate-phenyl-ester of approximately 20 µM (Ki value: 5.5 nM [16]).

### NA activity assay

Activity of expressed NA was measured based on published protocols [33], [34].

### Thrombin cleavage and purification of sulfated FLAG-tag

Purified non-FLAG reactive pN1/2009 NA was incubated overnight with 60 U of thrombin in carbonate buffer [50 mM] pH 8.0. Cleaved sulfo-peptide was purified via size exclusion chromatography (SEC; Superdex peptide; GE Healthcare) running in water. The purified fractions were subjected to mass spectrometry (MS).

### Mass spectrometry

MS was carried out as described elsewhere [17]. Briefly, SEC fractions were mixed 1∶1 with matrix solution (50 mM DHB in 1∶1∶1 water∶acetonitrile∶acetone) and 1 µl of the mixture spotted on to a ground steel target plate and allowed to air dry. MALDI TOF MS was performed in both positive and negative linear ion mode on an UltrafleXtreme MALDI TOF-TOF (Bruker Daltonics, Bremen, Germany). In negative ion mode, an acceleration voltage of 20 kV was used with an ion extraction delay of 80 ns while for positive ion mode the figures were 25 kV and 70 ns. Power settings were adjusted to optimize the signal intensity using the instrument software.

## Supporting Information

Figure S1 **Purification of non-FLAG reactive NA I.** FLAG-TB-NA (Hokkaido) depleted media was run over a lentil-lectin affinity column. The eluted protein was concentrated and subjected to gel filtration chromatography (Fig. S1A). Peak 1 consisted of soluble protein aggregates as shown in the corresponding SDS-PAGE (Figure S1B, fraction 7–9). Peak 2 was a mixture of two proteins (Fig. S1B, fraction 10–15). Fig. S1C shows the concentrated sample used for sulfatase assays or for further purification by anion exchange chromatography (See also Fig. S2). The higher molecular weight band was identified as Ecdysteroid UDP Glucosyltransferasse (EGT).
(TIF)

Figure S2 **Purification of non-FLAG reactive NA II.** FLAG-TB-NA (pN1/2009) depleted media was run over a lentil-lectin affinity column. Bound non-FLAG-reactive NA and co-purified EGT (see Fig. S1) were eluted, concentrated, and purified by gel filtration chromatography (Fig. S2A). Peak 1 contained soluble protein aggregates; Peak 2 consisted of non-FLAG reactive NA and EGT (see Fig. S1). The lectin-NA/EGT mixture was subjected to anion exchange chromatography. EGT eluted readily whereas lectin-TB-NA was eluted at higher salt concentration (Fig. S2B). Figure S2C shows the corresponding SDS-PAGE. Fractions 18–20 contained highly pure non-FLAG reactive TB-NA and were concentrated for subsequent experiments.
(TIF)

Figure S3 **Thrombin cleavage of Lectin-TB-NA.** Non-FLAG-reactive NA (pN1/2009; 1 µg/lane) was incubated in the absence and presence of H1 sulfatase (3 h, 37°C) and Thrombin (overnight, RT). After incubation all samples were subjected to anti-FLAG WB. Only a very weak signal was visible without sulfatase treatment whereas the addition of sulfatase restored reactivity of the FLAG epitope. Treatment with Thrombin abolished any signal with and without subsequent sulfatase incubation indicating that the FLAG tag was efficiently cleaved by Thrombin.
(TIF)

Figure S4 **Complete sequence of the expression constructs shown in** **Figure 1** **.** All constructs used for insect cell expression use the Melittin signal peptide (MSP, yellow) to drive secretion of the respective NA. The mammalian expression construct (D) uses a mouse Interleukin 3 (IL3; yellow) secretion signal. All constructs are based on an N-terminal FLAG tag (highlighted in blue) followed by an artificial tetramerization domain from yeast (A; GCN-pLI; highlighted in green) or Staphylothermus marinus (B, C, D; Tetrabrachion; highlighted in brown). Constructs A, B, and D were used to express Hokkaido H1N1 NA whereas Construct C is based on the sequence of pN1/2009.
(DOCX)
