# Supplementary figures and images for: Taking down the FLAG! How Insect Cell Expression Challenges an Established Tag-System
Source: PLoS One. 2012 Jun 6;7(6):e37779. doi: 10.1371/journal.pone.0037779 (PMC3368911; doi:10.1371/journal.pone.0037779)

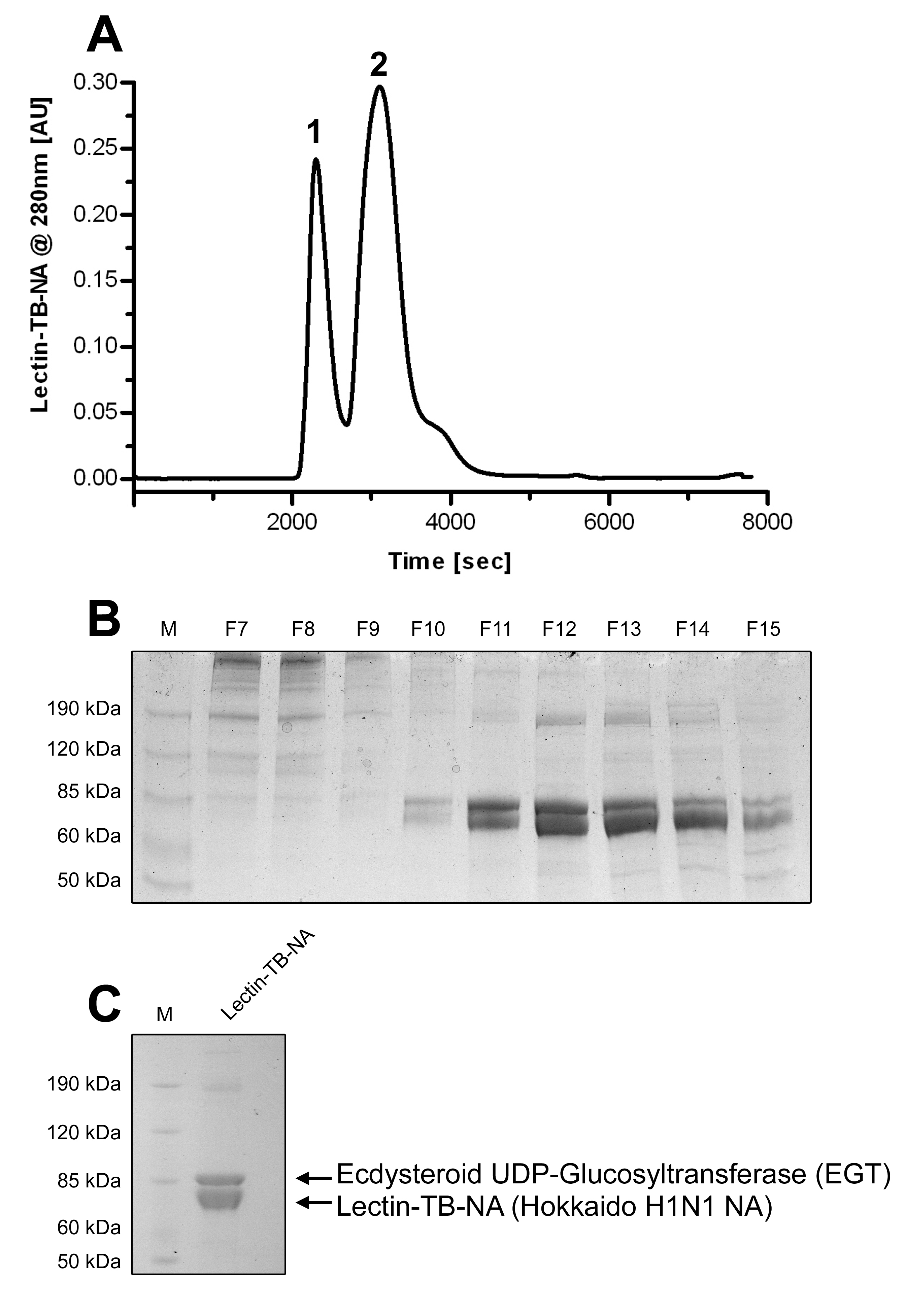

Supplement: Figure S1 — Purification of non-FLAG reactive NA I. FLAG-TB-NA (Hokkaido) depleted media was run over a lentil-lectin affinity column. The eluted protein was concentrated and subjected to gel filtration chromatography (Fig. S1A). Peak 1 consisted of soluble protein aggregates as shown in the corresponding SDS-PAGE (Figure S1B, fraction 7–9). Peak 2 was a mixture of two proteins (Fig. S1B, fraction 10–15). Fig. S1C shows the concentrated sample used for sulfatase assays or for further purification by anion exchange chromatography (See also Fig. S2). The higher molecular weight band was identified as Ecdysteroid UDP Glucosyltransferasse (EGT). (TIF) [file pone.0037779.s001.tif]

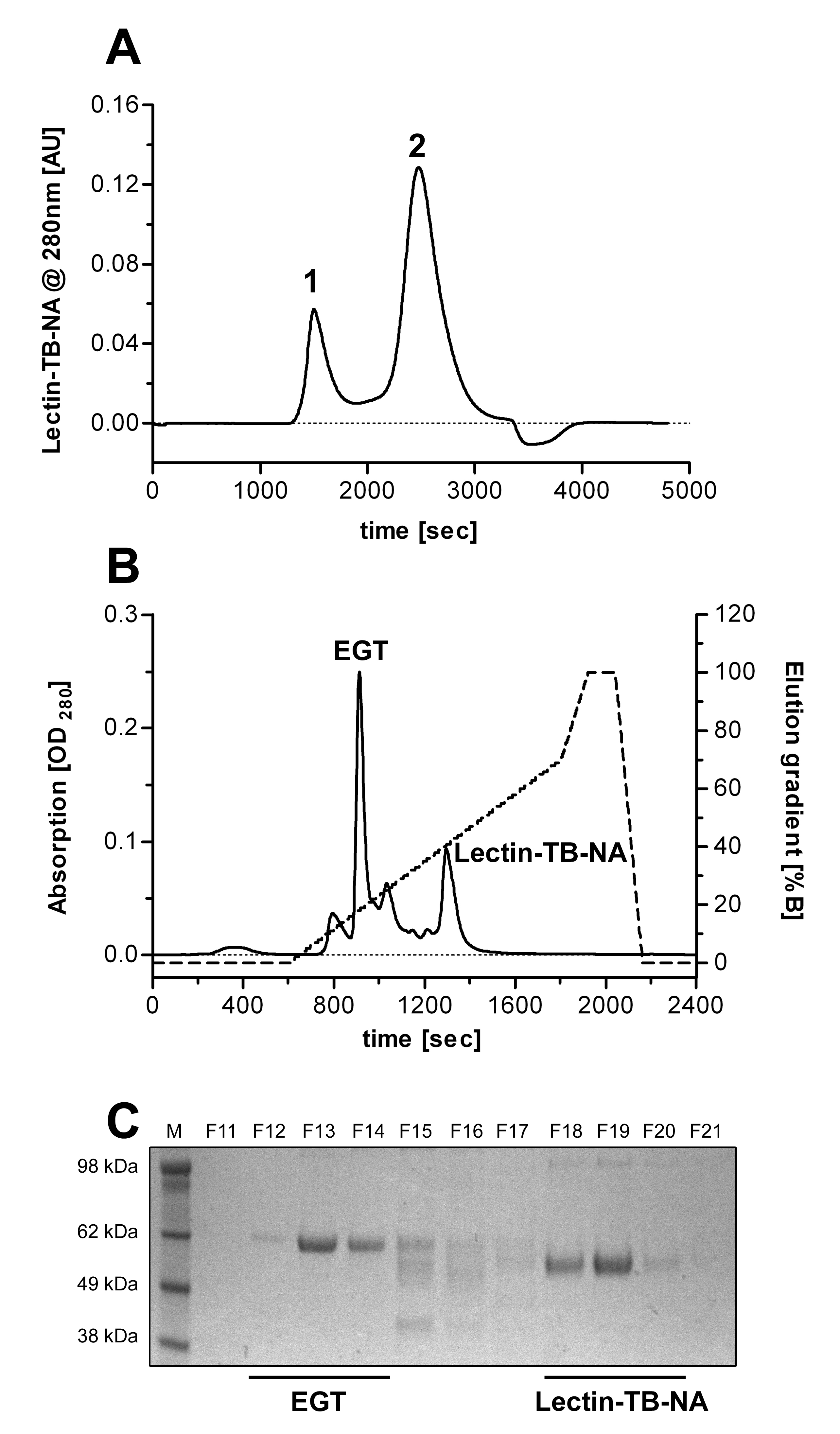

Supplement: Figure S2 — Purification of non-FLAG reactive NA II. FLAG-TB-NA (pN1/2009) depleted media was run over a lentil-lectin affinity column. Bound non-FLAG-reactive NA and co-purified EGT (see Fig. S1) were eluted, concentrated, and purified by gel filtration chromatography (Fig. S2A). Peak 1 contained soluble protein aggregates; Peak 2 consisted of non-FLAG reactive NA and EGT (see Fig. S1). The lectin-NA/EGT mixture was subjected to anion exchange chromatography. EGT eluted readily whereas lectin-TB-NA was eluted at higher salt concentration (Fig. S2B). Figure S2C shows the corresponding SDS-PAGE. Fractions 18–20 contained highly pure non-FLAG reactive TB-NA and were concentrated for subsequent experiments. (TIF) [file pone.0037779.s002.tif]

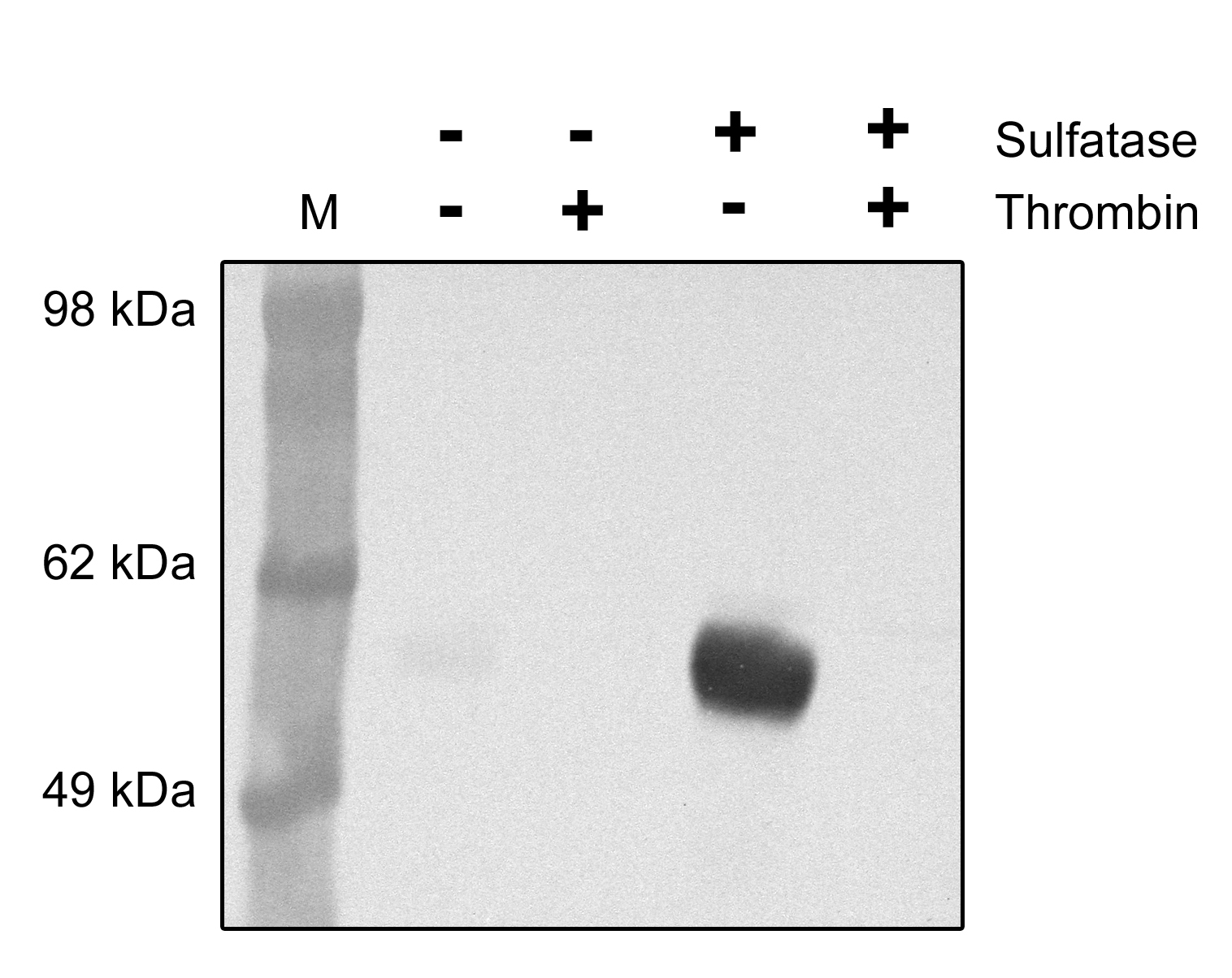

Supplement: Figure S3 — Thrombin cleavage of Lectin-TB-NA. Non-FLAG-reactive NA (pN1/2009; 1 µg/lane) was incubated in the absence and presence of H1 sulfatase (3 h, 37°C) and Thrombin (overnight, RT). After incubation all samples were subjected to anti-FLAG WB. Only a very weak signal was visible without sulfatase treatment whereas the addition of sulfatase restored reactivity of the FLAG epitope. Treatment with Thrombin abolished any signal with and without subsequent sulfatase incubation indicating that the FLAG tag was efficiently cleaved by Thrombin. (TIF) [file pone.0037779.s003.tif]
