# Supplementary material for: Taking down the FLAG! How Insect Cell Expression Challenges an Established Tag-System
Source: PLoS One. 2012 Jun 6;7(6):e37779. doi: 10.1371/journal.pone.0037779 (PMC3368911; doi:10.1371/journal.pone.0037779)
Supplement: Figure S4 — Complete sequence of the expression constructs shown in Figure 1 . All constructs used for insect cell expression use the Melittin signal peptide (MSP, yellow) to drive secretion of the respective NA. The mammalian expression construct (D) uses a mouse Interleukin 3 (IL3; yellow) secretion signal. All constructs are based on an N-terminal FLAG tag (highlighted in blue) followed by an artificial tetramerization domain from yeast (A; GCN-pLI; highlighted in green) or Staphylothermus marinus (B, C, D; Tetrabrachion; highlighted in brown). Constructs A, B, and D were used to express Hokkaido H1N1 NA whereas Construct C is based on the sequence of pN1/2009. (DOCX) [file pone.0037779.s004.docx]

**Construct A: Hokkaido N1 with MSP secretion signal (yellow), FLAG tag (blue), and Yeast GCN-pLI stalk (green) for expression in insect cells.**

**MKFLVNVALVFMVVYISYIYA**ef**DYKDDDDK**gg**lkqiedkleeilsklyhienelarikkllge**ggntnviagkdktsvtlagnsslcsisgwaiytkdnsirigskgdvfvirepfiscshlecrtffltqgallndkhsngtvkdrspyralmscplgeapspynskfesvawsasachdgmgwltigisgpdngavavlkyngiitetikswkkqilrtqesecvcvngscftimtdgpsngaasykifkiekgkvtksielnapnfhyeecscypdtgtvmcvcrdnwhgsnrpwvsfnqnldyqigyicsgvfgdnprpkdgegscnpvtvdgangvkgfsykygngvwigrtksnrlrkgfemiwdpngwtdtdsdfsvkqdvvaitdwsgysgsfvqhpeltgldcirpcfwvelvrglpkenttiwtsgssisfcgvnsdtanwswpdgaelpftidk

**Construct B: Hokkaido N1 with MSP secretion signal (yellow), FLAG tag (blue), and Tetrabrachion stalk (brown) for expression in insect cells.**

**MKFLVNVALVFMVVYISYIYA**ef**DYKDDDDK**gg**siinetaddivyrltviiddryeslknlitlradrlemiindnvstilasi**ggntnviagkdktsvtlagnsslcsisgwaiytkdnsirigskgdvfvirepfiscshlecrtffltqgallndkhsngtvkdrspyralmscplgeapspynskfesvawsasachdgmgwltigisgpdngavavlkyngiitetikswkkqilrtqesecvcvngscftimtdgpsngaasykifkiekgkvtksielnapnfhyeecscypdtgtvmcvcrdnwhgsnrpwvsfnqnldyqigyicsgvfgdnprpkdgegscnpvtvdgangvkgfsykygngvwigrtksnrlrkgfemiwdpngwtdtdsdfsvkqdvvaitdwsgysgsfvqhpeltgldcirpcfwvelvrglpkenttiwtsgssisfcgvnsdtanwswpdgaelpftidk

**Construct C: pN1/2009 N1 with MSP secretion signal (yellow), FLAG tag (blue), Thrombin cleavage site (red), and Tetrabrachion stalk (brown) for expression in insect cells.**

**MKFLVNVALVFMVVYISYIYA**m**dykddddklvprgg**g**siinetaddivyrltviiddryeslknlitlradrlemiindnvstilasi**ggntnfaagqsvvsvklagnsslcpvsgwaiyskdnsirigskgdvfvirepfiscsplecrtffltqgallndkhsngtikdrspyrtlmscpigevpspynsrfesvawsasachdginwltigisgpdngavavlkyngiitdtikswrnnilrtqesecacvngscftvmtdgpsdgqasykifriekgkivksvemnapnyhyeecscypdsseitcvcrdnwhgsnrpwvsfnqnleyqigyicsgifgdnprpndktgscgpvssngangvkgfsfkygngvwigrtksissrngfemiwdpngwtgtdnnfsikqdivginewsgysgsfvqhpeltgldcirpcfwvelirgrpkentiwtsgssisfcgvnsdtvgwswpdgaelpftidk

**Construct D: Hokkaido N1 with IL3 secretion signal (yellow), FLAG tag (blue), and Tetrabrachion stalk (brown) for expression in mammalian cells.**

**MVLASSTTSIHTMLLLLLMLFHLGLQASIS**ARQ**DYKDDDDK**gg**siinetaddivyrltviiddryeslknlitlradrlemiindnvstilasi**ggntnviagkdktsvtlagnsslcsisgwaiytkdnsirigskgdvfvirepfiscshlecrtffltqgallndkhsngtvkdrspyralmscplgeapspynskfesvawsasachdgmgwltigisgpdngavavlkyngiitetikswkkqilrtqesecvcvngscftimtdgpsngaasykifkiekgkvtksielnapnfhyeecscypdtgtvmcvcrdnwhgsnrpwvsfnqnldyqigyicsgvfgdnprpkdgegscnpvtvdgangvkgfsykygngvwigrtksnrlrkgfemiwdpngwtdtdsdfsvkqdvvaitdwsgysgsfvqhpeltgldcirpcfwvelvrglpkenttiwtsgssisfcgvnsdtanwswpdgaelpftidk
